# A systematic literature review of attitudes towards secondary use and sharing of health administrative and clinical trial data: a focus on consent

**DOI:** 10.1186/s13643-021-01663-z

**Published:** 2021-05-04

**Authors:** Elizabeth Hutchings, Max Loomes, Phyllis Butow, Frances M. Boyle

**Affiliations:** 1 Northern Clinical School, Faculty of Medicine, The University of Sydney, North Sydney, Australia; 2 School of Psychology, The University of Sydney, Sydney, NSW Australia; 3 Centre for Medical Psychology & Evidence-Based Decision-Making (CeMPED), Sydney, NSW Australia; 4 Psycho-Oncology Co-Operative Research Group (PoCoG), The University of Sydney, Sydney, NSW Australia; 5 Patricia Ritchie Centre for Cancer Care and Research, Mater Hospital, 25 Rocklands Road North Sydney, Sydney, NSW 2060 Australia

**Keywords:** Consent, Secondary data analysis, Data sharing, Attitudes, Healthcare consumers

## Abstract

**Background:**

We aimed to synthesise data on issues related to stakeholder perceptions of consent for the use of secondary data. To better understand the current literature available, we conducted a systematic literature review of healthcare consumer attitudes towards the secondary use and sharing of health administrative and clinical trial data.

**Methods:**

EMBASE/MEDLINE, Cochrane Library, PubMed, CINAHL, Informit Health Collection, PROSPERO Database of Systematic Reviews, PsycINFO and ProQuest databases were searched. Eligible articles included those reporting qualitative or quantitative original research and published in English. No restrictions were placed on publication dates, study design or disease setting. One author screened articles for eligibility and two authors were involved in the full-text review process. Conflicts were resolved by consensus. Quality and bias were assessed using the QualSyst criteria for qualitative studies.

**Results:**

This paper focuses on a subset of 47 articles identified from the wider search and focuses on the issue of consent. Issues related to privacy, trust and transparency, and attitudes of healthcare professionals and researchers to secondary use and sharing of data have been dealt with in previous publications. Studies included a total of 216,149 respondents. Results indicate that respondents are generally supportive of using health data for research, particularly if the data is de-identified or anonymised. The requirement by participants to obtain consent prior to the use of health data for research was not universal, nor is the requirement for this always supported by legislation. Many respondents believed that either no consent or being informed of the research, but not providing additional consent, were sufficient.

**Conclusions:**

These results indicate that individuals should be provided with information and choice about how their health data is used and, where feasible, a mechanism to opt-out should be provided. To increase the acceptability of using health data for research, health organisations and data custodians must provide individuals with concise information about data protection mechanisms and under what circumstances their data may be used and by whom.

**Systematic review registration:**

PROSPERO CRD42018110559 (update June 2020).

## Background

As healthcare moves to an increasingly digitised environment, new opportunities for researchers emerge. The use of health data traditionally collected for administrative purposes can be combined (linked) with other datasets to allow better insights into real-world clinical practice and patient outcomes. Further, this data can be used to inform health system design and responsiveness. In addition, data obtained during a clinical trial is also a key source of information; the secondary analysis of this data can confirm new findings and encourages transparency in research [1–4].

While the benefits of secondary data analysis have been widely discussed over the last decade, questions about how this data is accessed, by whom, and under what circumstances continue to attract debate. The principles of the International Conference on Harmonisation-Good Clinical Practice (ICH-GCP) and the requirement for obtaining informed consent prior to participating in research [5] are well known. However, the requirement for consent to use both administrative and clinical trial health data for secondary purposes is less clear to both patients and researchers. The use of this data, particularly health administrative data, is regulated by privacy laws of the country in which the data was collected. In Australia, the Commonwealth Privacy Act (1988) (the Act) [6] promotes and protects the privacy of individuals. Given the sensitive nature of health data, the Act provides extra protection around its collection and handling [7]. In certain circumstances, this data can be accessed for health and medical research, and where individual consent is impractical, two legally binding guidelines issued by the National Health and Medical Research Council (NHMRC) [7] add additional protections. The first stipulates data handling procedures for human research and ethics committees (HRECs) and researchers when using personal information disclosed from a Commonwealth agency for medical research (Guidelines under Section 95 of the Act) [7]. The second provides a framework for HRECs to assess proposals which use health information without an individuals’ consent (Guidelines under Section 95A of the Act) [7].

While there is a large amount of literature on the attitudes of healthcare consumers towards secondary data usage, it is fundamental that researchers develop a better understanding of the views of healthcare consumers towards consent for its use. Therefore, this paper presents a synthesis of health consumers’ attitudes towards consent and the use of administrative data and clinical trial data for research purposes.

## Methods

This systematic literature review presents the results of a subset of articles identified in a larger review of articles addressing data sharing which was undertaken in accordance with the PRISMA statement for systematic reviews and meta-analysis [8]. The protocol was prospectively registered on PROSPERO (www.crd.york.ac.uk/PROSPERO, CRD42018110559, updated June 2020). Given the substantial number of articles identified in our larger search, we have focused on specific issues over three articles; results relating to the attitudes of health researchers and issues relating to privacy, trust and transparency have been reported in other publications [9, 10].

The following databases were searched: EMBASE/MEDLINE, Cochrane Library, PubMed, CINAHL, Informit Health Collection, PROSPERO Database of Systematic Reviews, PsycINFO and ProQuest. The search was conducted on 24 June 2020. No date restrictions were placed on the search; key search terms are listed in Table 1.

**Table 1:** Example search strategy

PubMed	
**1**	((data sharing) OR (data link*) OR (secondary data analysis) OR (data reuse) OR (data mining))
**2**	((real world data) OR (clinical trial) OR (medical record*) OR (patient record*) OR (routine data) OR (administrative data))
**3**	attitud* OR view* OR opinion* OR perspective* OR satisfaction)
**4**	(patient* OR consumer*)
**5**	(doctor* OR clinician OR oncologist OR specialist*)
**6**	(Researcher* OR scientist* OR (data custodian*))
**7**	4 or 5 or 6
**8**	1 and 2 and 3
**9**	1 and 2 and 3 and 7

*Search includes ‘wildcards’ or truncation

Our original goal was to focus on attitudes towards data reuse by breast cancer patients. However, due to a paucity of studies targeting this group, we re-ran the search without this limitation and present the results of all disease settings. Breast cancer is a disease that impacts older individuals, therefore respondents under the age of 18 years were excluded from this analysis, as were attitudes towards biobanking and genetic research.

We note that increasingly the delineation between data collected for administrative purposes and other forms of electronic documentation such as electronic health records (EHR) (or other terms for these) becomes less clear. These records can contain both administrative and clinical data. Where possible, EHRs have been excluded from this literature review; however, we acknowledge that the lack of separation has made this a grey area.

Papers were considered eligible if they were published in English in a peer-reviewed journal; reported original research, were either qualitative or quantitative with any study design, related to data sharing in any disease setting; and included participants over 18 years of age. Reference list and hand searching were undertaken to identify additional papers. Systematic literature reviews were included in the wider search, but were not included in the results. Papers were considered ineligible if they focused on: electronic health records (including other terms for these); health information exchanges; biobanking and genetics; or were review articles; opinion pieces, articles, letters, editorials or non-peer-reviewed theses from masters or doctoral research. Duplicates were removed and title and abstract and full-text screening were undertaken using the Cochrane systematic literature review programme ‘Covidence’ [11]. One author screened articles for eligibility and two authors were involved in the full-text review process; conflicts were resolved by consensus.

Quality and bias were assessed at a study level using the validated QualSyst system for quantitative and qualitative studies as described by Kmet et al. [12]; the assessment was undertaken using the criteria for qualitative studies. No modifications were made to the QualSyst criteria prior to use. Quality and bias assessment was undertaken independently by two authors; conflicts were resolved by consensus. A maximum score of 20 is assigned to articles of high quality and low bias; the final QualSyst score is a proportion of the total with a possible score ranging from 0.0 to 1.0 [12].

Data extraction was by one author undertaken using a pre-piloted form in Microsoft Office Excel; a second author confirmed the data extraction. Conflicts were resolved by consensus. Data points included: author, country and year of study, study design and methodology, health setting and key themes and results. Where available, detailed information on research participants was extracted including age, sex, employment status, highest level of education and health status.

Quantitative data were summarised using descriptive statistics. Synthesis of qualitative findings used a meta-aggregative approach, in accordance with guidelines from Lockwood et al. [13]. The main themes of each qualitative study were first identified and then combined, if relevant, into categories of commonality. Using a constant comparative approach, higher order themes and subthemes were developed. Quantitative data relevant to each theme were then incorporated. Using a framework analysis approach as described by Gale et al. [14], the perspectives of different groups towards data sharing were identified. Where differences occurred, they are highlighted in the results. Similarly, where systematic differences according to other characteristics (such as age or sex) occurred, these are highlighted.

## Results

**Fig. 1:**
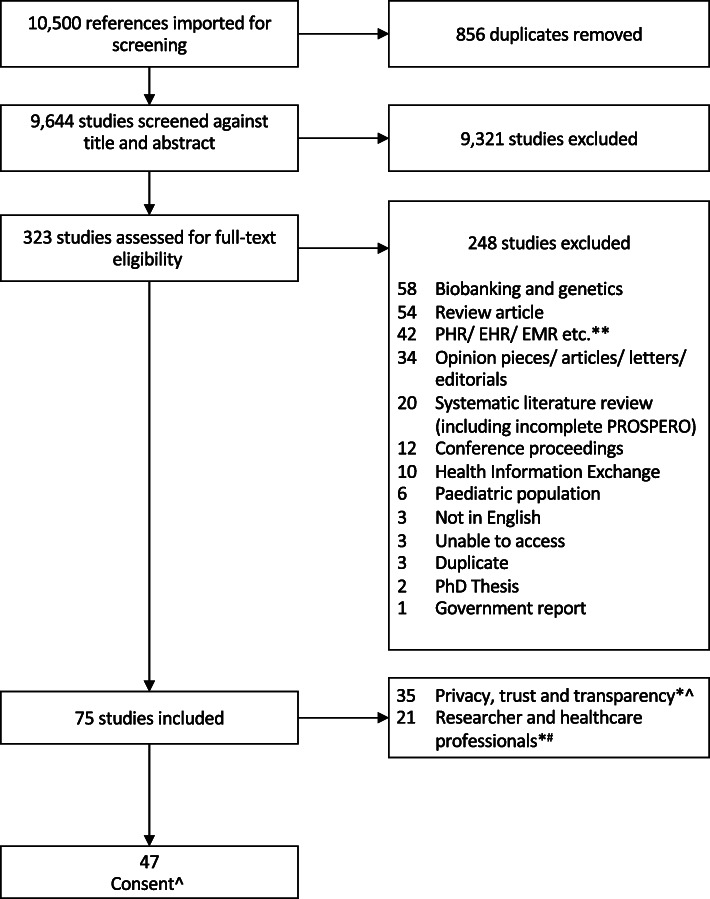
PRISMA flow diagram

This search identified 10,500 articles, of which 323 underwent full-text screening; 75 articles met the inclusion criteria for the larger review. The PRISMA diagram is presented in Fig. 1. This article presents a subset of the results of the wider search and focuses on issues of consent.

A subset of 47 [15–61] of the 75 articles addressed issues relating to consent in the context of sharing health data and were included in this analysis (Fig. 1 and Table 2). A total of 216,149 respondents were included in the studies.

**Table 2:** Included studies

Author, location, date of data collection	Methodology, sampling, analysis	Health condition/setting	No. of participants (***N***)	Participant demographics ***n*** (%)	Outcomes, result(s)	QualSyst score
**Quantitative**						
[15], Canada, October 2015 and November 2016	Survey, mean (SD or IQR) and Fisher exact test	General medicine /cardiology patients	590	**Age, median** 65, IQR 8 **Sex, male** 420 (71) **Education** 27 (4.6), primary school 101 (17.1), did not complete HS 144 (24.4), HS 77 (13), professional training 142 (24), University or college 77 (13), Masters or PhD 23 (3.8), prefer not to disclose **Ethnicity** 535 (91.6), Caucasian 15 (3.6), Black & Caribbean 3 (0.5), First nations 12 (2.0), Asian 5 (0.8), Other 17 (2.9), prefer not to disclose	97.4% allowed researchers access to their record in the administrative database for the purposes of conducting a clinical trial. 75.3% would allow the use of their initials and DOB to link administrative record to their medical charts. 53.7 preferred the use of administrative records for follow-up care compared with 30.9% for face-to-face appointments.	0.9
[17], USA, not reported	Survey, not reported	General medicine/patients with asthma or stable angina	3429	No reported	335 (9.8) refused consent to data collection from their medical records for research. Information relating to stable angina or asthma was not considered particularly sensitive. Consent rates were not dissimilar by disease, sex, mean age, severity of symptoms, or satisfaction with care.	0.35
[18], USA, not reported	Survey, logistic regression	General medicine/general public	2271	Not reported	96.3% consented to data linkage. Only two variables were significant: government insurance (OR = .332[.442–.775], *p* = .011) and health literacy (OR = 1.35[1.09–1.67], *p* = .006). Those with government insurance were less likely to consent to data linkage of survey data with clinical data.	0.25
[20], USA, not reported	Survey or telephone interview, logistic regression	General medicine/general public	1574	**Age, years** 232 (15), 18– < 35 512 (33), 35– < 50 465 (30), 50– < 65 365 (23), 65+ **Sex, male** 794 (50.4)	86.2% of respondents authorised data linkage of survey and health administrative data. Non-authorisers (*n* = 217) were significantly younger (born between 1975 and 1993), had less comorbidities, and were less likely to have visited clinic (< 3 visits) in one year.	0.9
[21], Australia, April and June 2016	Survey, absolute numbers and frequency, Fisher exact test and ordinal logistic regression	General medicine/hospitalised patients	919	**Age, years** 177 (19), 18–30 287 (31), 31–50 261 (28), 51–70 158 (17), 71–85 36 (4), > 85 **Education** 180 (20), did not complete HS 266 (29), HS 192 (21), vocational 206 (22), bachelor’s 75 (8), master’s/doctoral **Sex, male** 406 (44) **Primary language, English** 616 (67)	421 (46) assumed that their data was already used for medical research (without explicit consent). 810 (88) approved or were indifferent to their medical data used for treatment purposes to be reused for research purposes. 800 (87), found opt-out consent acceptable or were indifferent. Preferred consent models • 494 (54), opt-out • 172 (19), no preference • 253 (28), opt-in • Sex, employment status, and cognitive status did not impact the acceptability of opt-out consent. • Opt-out had higher rates of support in refugee (93%) and those who were illiterate in their primary language (92%). To withdraw consent: 431 (40) preferred a telephone voicemail message and 330 (30) preferred email correspondence.	0.65
[23], Britain, October to December 2004	Questionnaire, raw figures with percentages	Recently discharged medical and surgical patients/hospital	166	Not reported	21% *definitely wanted* or *preferred* to be asked for permission to use anonymised medical history and 20% for reasons for treatment. The proposed use of information (e.g., research, audit) made little difference to the overall approval percentages (range 10–12%). 13% *definitely wanted* to be asked for permission for use of information; most had no preference or preferred not to be asked. The most popular method for asking was signing a form while in hospital, rather than a request later. Being informed each time material was used, but not being asked for your permission, was the second most popular option. • 7% and 5% wanted to be asked permission when their age or gender was required respectively. 14% wanted to be asked when sexual orientation was asked. • 10% wanted to be asked if information was to be used for clinical audit. • ~ 10% preferred to be asked for permission when information was intended to help future patients or to teach healthcare professionals. • 12% wanted or preferred to be asked permission when being used for publication in medical journals.	0.9
[25], Europe, March to May 2018	Survey, descriptive statistics, and Chi squared test for independence	Rare diseases/patients and their families with rare diseases	2013	**Age, years** 2 (0), 15–17 70 (3), 18–24 293 (15), 25–34 852 (42), 35–49 644 (32), 50–64 152 (8), ≥ 65 **Sex, male** 473 (23) **Location** 1775 (88), EU 238 (12), non-EU **Diagnostic status** 1909 (95), diagnosed 104 (5), undiagnosed **Number of rare disease (*n*** **= 1909)** 1664 (87), 1 174 (9), 2 44 (2), 3 13 (1), 4 14 (1), ≥5	Reasons for consenting to research using respondent’s health data included: development of new treatments (97%), better understanding of their disease (97%), improved diagnosis (97%), receiving additional specialist advice (95%), and improved research and care for their disease (90%). • Only 51% consented when data was to be used for research not related to the medical field. Respondents over 65 years of age were less willing to consent to share data for research on other diseases (84%). Respondents with lower levels of education were more willing to share (59%) compared with those with higher levels of education (48%). Disease severity—those with more severe rare disease were more likely to share data (64%) compared with those with less severe disease (40%). Incentives to increase participation included: more information about the disease, be informed of the results of research, capacity to withdraw from the research, and being informed of any data breaches. Perceived sensitivity of data (disability, genetic information, physiological data, symptoms, name of disease) all included the rates of consent. • 51% believe that information on disability was sensitive, the lowest rate was seen for the name of the disease (33%). Willingness to share data is not precluded by wanting to control access. • Only 1% did not request any control over their data; 47% wanted full control on access. • Women were more likely to request control (48%) compared with men (42%). Reasons for not consenting included: fear of discrimination (34%), fraud (32%), concerns about personal safety (20%), use of data without knowing (35%), and used in a context that they do not approve (47%). • 50% of respondents wanted control to restrict access to third parties. 49% were willing to allow an ethics committee to decide about access on their behalf; 43% were against this. Communication is essential and should include how the data is to be used, and the results of this research.	0.95
[26], UK, January 2009 to December 2010	Panel survey, descriptive analysis, *t*-test, logistic regression	General medicine/general public	50,994	**Age, years** 6897 (16.3), 16–24^ 7495 (17.7), 25–34 8300 (19.6), 35–44 7846 (18.6), 45–54 6809 (16.1), 55–64 4959 (11.7), 64–74 **Education** 9819 (23.2), none 18, 744 (44.3), secondary level 13,745 (32.5), higher/degree **Sex, male** 20,992 (49.6)	70.7% of respondents consented to record linkage. 75% of respondents age 16–24 years consented. • Similar proportions 71% (males) and 70% (females) consented to data linkage. • Younger age, marriage, employment status, car ownership, and education were all significantly associated with consent. Small increases in consent rates in individuals with poor health (self-assessed) [adjusted OR 51.11; 95% CIs: 1.06, 1.16], less so when defined by General Health Questionnaire score (adjusted OR 5 1.05; 95% CIs: 1.00, 1.10). Larger differences were observed for those of non-white ethnicity who were 38% less likely to consent (adjusted OR 50.62; 95%CIs: 0.59, 0.66). Consent was higher in Scotland than England (adjusted OR 5 1.17; 95% CIs: 1.06, 1.29) but lower in Northern Ireland (adjusted OR 5 0.56; 95% CIs: 0.50, 0.63).	0.95
[28], Europe, 2012	Questionnaire, results expressed in percentages	Leukodystrophy/leukodystrophy patients and family	195 (149, family; 46, patients)	**Age, years** 121 (62), 40–64 **Country** 130 (66.7), France 24 (12.3), Italy 9 (4.6), Belgium 6 (3.1), Spain 26 (13.3), Germany	Patients and their families were strongly in favour of participating in the registry. A process combining initial broad consent with ongoing information was appropriate. Information should be given to the patient at initial consent.	0.85
[29], UK, 1996–2000	Survey (seven), random effects model	Epidemiological research/general public	27,797	**Age, years** 2396 (8.6), 18–29 1792 (6.4), 30–39 2317 (8.3), 40–49 7714 (27.6), 50–59 6824 (24.5), 60–69 5116 (18.4), 70–79 1838 (6.6), 80+ **Sex, male** 11,476 (41.3)	Responders < 50 years of age 75–95% gave consent to follow-up. This fell in older people, particularly females. In one study consent fell to < 50% in women ≥ 80 years of age. Males, younger people, and subjects reporting the symptom under investigation were more likely to give consent. • Responders who had the symptom being investigated had ~ 1.5 times the odds of consenting to review of their medical records as those not reporting symptoms.	1
[30], Finland, not reported	Survey, descriptive statistics	General medicine/general public	418	**Age, years** 44 (10.5), ≤ 30 47 (11.2), 31–40 57 (13.6), 41–50 88 (21.1), 51–60 126 (30.1), 61–70 49 (11.7), > 70 7 (1.7), missing **Education** 74 (17.7), PS 44 (10.5), SS 165 (39.5), HS 80 (19.1), University of Applied Sciences or bachelor’s degree 52 (12.4), Master’s degree or higher 3 (0.7), missing	83% had a positive or very positive opinion about health research in general. 49% (*positive*) or 19% (*very positive*) opinion about using administrative health registries for research purposes. 3% had a *negative* or *very negative* opinion. 61% were willing to provide identifiable health information for research. • 8% would like to be informed when their information is used. • 86% favoured broader consent methods - one consent covering a certain register or a research topic. • 55% required ethical evaluation from register-based research addressing a sensitive issue. When asked about Finnish legislation, 35% of the study population wanted to tighten the law for some parts, 28% were satisfied with the current practices, and 25% wanted to liberalize the law to advance scientific research. Supported research purposes for health registries included: • 76% aetiologic studies. • 60% disease monitoring. • 53% assessing the effectiveness of health care. • 38% any research use. *Register-based research* • 48% (positive) and 12% (very positive) towards the use of their own health information in register-based research. • Respondents with higher education were more often inclined than those with a lower education level to allow. Of the participants with the highest education level, 82% had a positive or very positive opinion compared with participants with the lowest education level (41%) (*p* < 0.001). *Use of hospital medical records* • 75% would like to be informed of the possible research use of their medical records on admission to hospital. • 52% would like to be able to limit research use of their medical records. • 11% forbid it altogether. • 29% thought that everyone’s medical records should be accessible for research. *Register linkage* • 32% would like to be able to limit linkage of certain registries. • 34% felt that everyone’s information should be available for researchers in every national register. • 15% would forbid record linkage altogether. • 30% indicated no need for informed consent in register-based research. • 30% similar proportion felt consent should be obtained for every study. • 39% thought consent necessary, in some situations, such as studies addressing a sensitive study topic. • Men found informed consent unnecessary more often than women (37 % vs. 27 %), whereas women more frequently thought that in special cases informed consent should be required (44 % vs. 31 %).	0.85
[32], Canada, August 2014 and May 2015	Survey, descriptive statistics, and Student’s *t*-tests and nonparametric tests	Cancer/outpatient clinic	569	**Age, years** 59, median **Cancer type** 109 (19.2), breast 86 (15.1), gastrointestinal 83 (14.6) genitourinary 70 (12.3), thoracic 73 (12.8), hematologic 73 (12.8), head and neck 63 (11.1), gynaecologic 12 (2.1), other **Clinical trial participation, yes** 183 (32.2) **Education** 346 (60.8), university, college, professional 39 (6.9), vocational, technical, diploma 169 (29.7), elementary, HS 15 (2.6), prefer not to answer or missing **Ethnicity** 452 (79.4), white 68 (12), Asian 49 (8.6), other/prefer not to answer, missing **Sex, male** 234 (41)	93%, (cohort 1) would allow long-term access to their information and allow personal information to be used to match clinical trial with administrative data. 68% (cohort 2) preferred to make additional clinical information available through linkage with administrative databases. 9% preferred to have no further information made available to researchers. No significant differences were found in the subset of patients who were part of a clinical trial compared with those who had never participated (p = 0.65). Canadian Primary Care Sentinel Surveillance Network has established policy governing the protection of privacy and use of health information for research where no individual patient consent is required.	0.75
[34], Taiwan, 2001	Survey, multiple logic regression	General medicine/general population	14,611	**Age, years** 1769 (12.1), 20–24 3263 (22.3), 25–34 3436 (23.5), 35–44 2782 (19), 45–54 1606 (11), 55–64 1214 (8.3), 65–74 541 (3.7), 75+ **Education** 3776 (25.8), ≤ college 4366 (29.9), senior high 2266 (15.5), junior high 2942 (20.1), elementary 1261 (8.3), illiterate **Sex, male** 7195 (49.2)	2911 (88%) gave consent to link their questionnaire to their NHI records. Age over ≤ 65 years, married, illiterate, those with a monthly household income < 30,000 New Taiwan (NT) dollars, or were living in a suburban area were less likely to consent. • Non-consenters had relatively lower mean scores in all eight physical and mental functional status domains of SF-36. No difference in gender and self-reported health was between individuals who consented and those who refused was noted.	0.85
[37], UK, September to December 2009	Survey, bivariate associations and estimate multivariate logistic regression	General medicine/BHPS Wave 18	13,454	**Age, years** 1965 (14.6), 16–24^ 3200 (23.8), 25–39 2546 (18.9), 40–49 1984 (14.7), 50–59 3758 (27.9), ≥ 60 **Country** 6633 (49.3), England 2357 (17.5), Wales 2282 (17), Scotland 2182 (16.2), Northern Ireland **Education** 425 (3.2), higher degree 1593 (11.8), first degree 4220 (31.4), diploma in higher education 1711 (12.7), A-levels 2130 (15.8), O-level or equivalent 663 (4.9), GCSE 67 (0.5), Commercial qualification, no O-level/GCSE 2395 (17.8), none/still at school **Ethnicity** 12,077 (89.8), British/Irish White 1377 (10.2), other **Health problems related to:** *(note, not all reported)* 1793 (13.2), chest 2528 (18.8), heart 1046 (7.8), stomach 642 (4.8), diabetes 1119 (8.3), anxiety 208 (1.5), cancer **Sex, Male** 6069 (45.1)	*Note: Additional data taken from publication supplementary tables* 41% consented to health data linkage. • 99% of those who gave consent to link to health data to the BHPS also gave consent to link to the NHS Central Register. Consent was significantly higher among people who live in: • England (42.4%) • Participants aged 16–24 (45.9%) • Participants who considered their ethnicity to be British/Irish White (42.4%). Consent by education (two highest, two lowest): • 49.2%, higher degree. • 45.6% A level. • 36.8%, none/still at school. • 21.7%, commercial qualification, no O-level/GCSE. Consent was not affected by socio-economic or health characteristics. Patients with cancer consented 46.2% of the time. Difference by sex: Male, 42%; Female, 40.7%. Recent users of GP services were underrepresented among consenters. Self-reported health was generally not associated with consent. There are two exceptions; diabetes and obesity which were associated with rates of consent.	0.9
[40], USA, not reported	Survey, percentage of respondents	General medicine/patients with DM and congestive heart failure	4647	*Participants at sites where no advanced permission required reported (****n*** **=** *1174)* **Age, mean** 65.9 **Condition** 587 (50), congestive heart failure **Sex, male** 587 (50)	Type of review required: • 10 sites required full IRB review prior to patient contact. • 1 site had an expedited IRB review. • 4 sites deferred to the IRB at RAND Health. Type of consent required: • 8 sites required no advance permission to contact the potential participants for a telephone survey. • 5 sites required oral permission. • 2 sites required written advance permission. Overall, the contact rates and eligibility rates were similar across different sites. Response rate varied based on type of consent process; this was highest for sites requiring no advanced permission to contact potential study participants. *Sites without advance permission requirements* • 85% of eligible participants consented to the telephone survey. • Participation rates were highest for sites not requiring advanced permission. • 58% of participants at these sites completed the telephone survey compared with 39% at sites requiring oral advanced permission and 27% from sites requiring advanced written permission. *Sites requiring permission* • The sites with written advanced permission had the lowest overall cooperation rate of 39%, with only 43% of potential participants providing permission for researchers to access their contact details. Patients with congestive heart failure were slightly more likely to complete the survey than individuals with diabetes.	0.95
[41], Hong Kong, not reported	RCT nested within a cohort, chi-square test, multivariable logistic regression, likelihood ratio test	General medicine/subsample of the FAMILY cohort	1200	**Age, years** 94 (7.8), 18–29 197 (16.4), 30–44 423 (35.3), 45–59 307 (25.6), 60–74 179 (14.9), ≥ 75 **Education** 436 (36.3), primary 587 (48.9), secondary 177 (14.8), tertiary **Sex, male** 456 (38)	33.3% of respondents returned signed consent forms. Subgroup analyses found requesting HKID significantly reduced consent among adults aged 18–44 years of age (OR 0.53, 95% CI 0.30–0.94, compared with no request). Souvenir incentives increased consent among women (OR1.55, 95%CI 1.13–2.11, compared with no souvenirs), but no overall effects were noted. Younger people also had increased rates of consent with a souvenir. Higher income and older age were associated with health record linkage. The request for a unique personal identifier did not substantially reduce consent proportions.	0.95
[42], Canada, November 2003	Survey, descriptive statistics, multiple logistic regression.	AIDS, MS, mental health/outpatients	235	**Age, years** 68 (28.9), 20–39 129 (54.9), 40–59 34 (14.5), ≥ 60 4 (1.7), unknown **Education** 39 (16.6), < grade 12 35 (14.9), grade 12 156 (66.4), attended/finished post-secondary 5 (2.1), no answer **Previous experience with medical research, yes** 127 (54) **Sex, male** 86 (36.6)	Respondents believed that the physician involved in their care should be the only ones who should be able to access their health data without consent. • Over 78% of respondents believed that consent was required when the data is identifiable. • Over 84% of respondents believed that consent was required when the data is of a sensitive nature. • 17% were not sure about seeking consent when it was not feasible. • 63% believed that consent was not necessary when the information was anonymous. Access to health information without consent: • 33.2%, medical researchers. • 4.3%, drug companies. • 4.3%, employer. • 8.1%, insurance company. • 6%, the government. Overall, 78.3% were advocates for consent. The difference in patient groups was not significant. • Sex and employment status were the only factors to predict consent. Women (OR = 1.96, 95% CI: 1.04–3.71) and those who were employed (OR = 2.29, 95% CI: 1.00–5.25) were both advocates for consent. • 77.4% agreed/strongly agreed that researchers should get consent when the data was identifiable. • 22.5% agreed/strongly agreed with one of consent for all future medical research. • 38.3% agreed/strongly agreed that people should be informed about their health information being used (no additional consent required). • 77.2% agreed/strongly agreed that consent is required for each new project. • 34% agreed/strongly agreed that researchers should be able to use un-identifiable information without consent.	0.85
[44], Northern Ireland, September to December 2015	NILT survey, univariate and multivariate analyses	General medicine/general public	1202	*Results of weighted demographics* **Age, years** 144 (12), 18–24 175 (14.6), 25–34 172 (14.3), 35–44 214 (17.8), 45–54 180 (15.0), 55–64 310 (25.8), ≥ 65 7 (0.6), not answered/refused **Education** 224 (18.6), no qualification 555 (46.2), school level 369 (30.7), graduate level 54 (4.5), not answered/refused **Sex, male** 559 (46.5)	Respondents believed that academic researchers should be allowed to use data that has been linked by a third party. • When the data is linked by the NHS—88% (males) and 86% (females) responded *defiantly* or *probably should* be allowed. • 10% had problems with NHS linking data. • When the linking was undertaken by researchers and the data included postcodes—63% (males) and 58% (females) responded *defiantly* or *probably should* be allowed. • When the linking of data is undertaken by people who keep hospital records and linked to school records—74% (males) and 71% (females) responded yes (*probably* or *defiantly*). Those with a long-term health condition were more likely to be against data sharing (24–25%) compared with those with no long-term health condition (20%). Respondents were divided on the issue of consent for linked data analysis. • 30% believed that it was not necessary to ask for consent to link data if individuals will not be identified. • 34% believed that consent should always be sought but ‘if the difficulties are too great, important research should not have to be abandoned for this reason’. • 31% of respondents believed that data should only be used with individual patient consent before linking the data with anything else. If consent was not possible this may mean not undertaking the research. • By sex, 34% of males and 29% of females agree with this statement. • Those with no qualifications were more likely not to allow research without consent (38%, no qualification to 26% with graduate=level qualifications). • 30% of respondents with no health condition, 15% of respondents with a health condition (ADLs not affected), and 42% (ADLs reduced a little) and 40% (ADLs reduced a lot) would require consent. • 5% did not know if consent was needed to link data. Connection between the requirement to get consent to use data and trust in the organisation to secure the data. • Those who felt that research should not be done without consent, were more likely to state that they did not trust the NHS (20% vs 9%), the government (35% vs 20%), GP practices (11% vs 5%), commercial organisations (63% vs 54%) and academics (29% vs 19%).	0.9
[46], UK, 2009	BHPS Wave 18, multivariate bivariate probit models	General medicine/BHPS Wave 18 participants	6433	Not reported	In the UK consent must be obtained to link administrative data. 41% of respondents gave consent for health data linkage; 32% consented to the linkage of benefit (economic) records; 39% agreed to link to education records. Consent to data linkage was related to the respondent’s views on privacy and community-mindedness. • Males were more likely to consent to health and benefit data linkage. • Respondents aged above 24 years of age were less likely to consent; however, this was not statistically significant. • Socio-economic variables did not influence consent. • Respondents with higher levels of education were more likely to consent. • Refusal to answer questions on income from investment was a strong predictor of not giving consent. • Undertaking voluntary work was associated with giving consent for health records access. The probability to consent is associated with the number of household members who have already consented to health and benefit data linkage.	0.65
[47], UK, not reported	Postal survey, randomised factorial design	General medicine/general public	245	**Age, years** Between 65 and 74	86.9% of respondents gave consent to access the medical records. The inclusion of an income question or seeking to access medical records did not reduce the response rates in older people. • 75.8% of respondents answered the income question.	0.45
[48], Australia, 1999	Survey (mail), chi squared with Yates correction	General medicine/university workers/those with upper body and neck disorders	292 (200—women employed by a university; 92—women with upper body and neck disorders)	**Age, years** 184 (63), ≤ 45 **Education** 158 (54), secondary education **Sex, Male** 0 (0)	*Participants were randomised 1:1 to receive a postal questionnaire plus or minus an authorisation form (the other group received the authorisation form later).* 38% (university workers) and 24% (patients) provided authorisation after receiving the authorisation form later, compared with 31% and 17% in those who received the form at the same time. • Differences in authorisation rates may be due to the sensitive nature of data for the patient group. A delay in seeking approval to link data was associated with improved survey response rates while not impacting on authorisation rates.	0.65
[50], Germany, 2011 and 2014	Computer assisted personal interviews, binary logistic regression	General medicine/lidA study participants	4148	**Year of birth**^**#**^ 1871 (45.1) 2277 (54.9) **Education** 1000 (24.1), low level 1767 (42.6), medium level 1381 (33.3), high level **Health status, subjective** 2240 (54), very good to good 1336 (32.2), satisfactory 572 (13.8), less good to poor **Migrant background** 4015 (96.8), born in Germany **Multimorbidity** 718 (17.3), no disease 983 (23.7), 1 disease 917 (22.1), 2 diseases 1535 (37), ≥ 3 diseases **Sex, male** 1879 (45.3)	93.8% of respondents gave consent to link primary data with at least one other data source (administrative or claims data). • 0.8% (*n* = 30) SHI data only. • 22.1% (*n* = 867) IAB data only. • 77.1% (*n* = 3021) IAB and SHI data. Respondents from the former West Germany (excluding West Berlin) were less likely to have their study data linked with both data sources or with IAB data only compared with respondents from the former East Germany and Berlin. Those with technical or advanced technical school were less likely to agree to link their primary data with IAB data only than those with apprenticeship or school-based vocational training. Those with no apprenticeship or with a non-regular apprenticeship were more likely to refuse consent completely. Respondents who gave no information on income were more likely to refuse consent to both IAB and SHI data linkage. If they did give consent it was predominantly for IAB data only. Respondents’ health status influenced levels of consent. Respondents with two or three or more diseases agreed more frequently to both data sources. • Subjective health (SF-12v2) did not impact the levels of consent.	0.95
[54], New Zealand, not reported	Survey, chi-square tests	General medicine/general public	203	**Age, years** 106 (56), 18–34 69 (37), 31–-60 14 (7), ≥ 61 **Ethnicity** 146 (72), New Zealand Europeans 55 (27), Maori **Sex, male** 61 (32)	Respondents were more willing to share their information if it was de-identified. • 60% of respondents had some concerns about sharing anonymous data with people other than HCPs. Responses were influenced by the data recipient and the nature of the information. Respondents were willing to consider data sharing all their health information with HCPs if consulted. Few refused to share their information. • Over 50% of respondents did not wish to share data with government agencies or health insurers. Respondents were less likely to share data of a sensitive nature, with those least involved in their care. Implied consent was not always well-informed consent; patients should be made aware of the current data-sharing practices. Use of a hybrid model of consent—general consent with specific denial may be appropriate to access data within the clinical setting, and general denial with specific consent to access data for other purposes. • It is unclear if this consent would be for a single episode or more general access. • The method for obtaining consent varies based on the proposed use of data.	0.7
[55], Canada, March to April 2005	Survey, response frequencies	General medicine/general public	1230	**Age, years** 480 (39), 18–39 504 (41), 40–59 246 (20), ≥ 60 **Education** 406 (33), HS or less 172 (14), some postsecondary 492 (40), completed postsecondary 123 (10), postgraduate or professional degree **Sex, male** 554 (45)	Access to personal information for health research. • 4% of respondents did not believe that information in their paper medical records should be used for medical research. • 32% believed that permission should be obtained for each use. • 29% supported broad consent. • 24% supported notification and opt-out consent. • 11% believed that no need for notification or consent. 80% of those willing to give general permission wanted to be able to periodically review this decision. • 36% wanted no or minimal involvement. • 24% satisfied with notification • Opportunity to opt-out 43% very important and 46% somewhat important. • 12% acceptable to use with no notification or permission. Respondents preferred data to be extracted by a nurse at the doctor’s office, but not the secretary. A research assistant from the university was also acceptable. • 20% were not happy with this option. 70% of respondents supported the introduction of a common EMR. • 9% believed that this data should not be used at all; this is higher than that for paper records (4%). • 27% believed that EMR data could be used without permission (compared with 12% for paper records). 27% of respondents were against linking income data with EMR records. • 16% supported notification • 17% supported use without permission or notification.	1
[58], USA, not reported	Survey, t test and multivariate logistic regression	General medicine/general public	1106	**Age, mean** 41 years **Sex, Male** (22) **Education** (55), HS or higher education	67% of respondents provided consent to access data for health services research; 8% did not answer (passive non-consenter). • 25% actively refused. Those who consented were older and included fewer women and African Americans than those who consented. Difference between non-consenters and those who passively non-consenters were noted. • Non-responders were older, less educated, lower income, and included more African Americans compared with those who actively refused consent. Respondents who actively consented were younger, included more women and were more educated compared with those who consented. Respondents who consented had a significantly lower PCS compared with those who did not consent. • Those with the highest physical functioning scores were more likely to refuse consent (significant). Non-consent rates were highest in respondents seeking: contraception (46.7%), treatment of urinary disorders (39.4%), uncomplicated DM (37.5%), headache (37%), and female genital disorders (35.4%). Severity of illness did not impact the rates of consent between groups. Respondents were less likely to give consent if they did not answer questions on smoking status, income, or functional status.	1
**Qualitative**						
[16], UK, not reported	Interviews, thematic analysis was undertaken using the Framework approach	General medicine/individuals included in the ALSPAC birth cohort study	55	**Age, years** 12 (21.8), 17^ 35 (63.6), 18 8 (14.5), 19 **Education** 7 (12.7), at university 25 (45.5), A-levels 8 (14.5), GCSE’s 12 (12.8), other 3 (5.45), none **Ethnicity** 51 (92.7), white British 3 (5.5), other 1 (1.8), refused **Health status, self-reported** 9 (16.4), disability/long term illness 46 (83.6), no disability/long term illness **Sex, male** 24 (43.6)	Some respondents were unsure of the effectiveness of data anonymisation and therefore did not believe that consent was unnecessary. Participants equated consent with opt-in and being asked if their data could be used for a specific study. No consensus was reached for any of the scenarios for consent. 1. Linking teenage pregnancy data with state benefits: • One suggested that this study not take place (*n* = 1). • Unclear/unsure (*n* = 11). • Request consent (*n* = 34). • No consent required (*n* = 9). 2. Linking birthweight to future health outcomes: • Unclear/unsure (*n* = 8). • Request consent (*n* = 36). • No consent required (*n* = 11). 3. Linking mental health records and criminal records: • Unclear/unsure (*n* = 15). • Request consent (*n* = 20). • No consent required (*n* = 11). 4. Linking asthma and postcodes: • Unclear/unsure (*n* = 15). • Request consent (*n* = 26). • No consent required (*n* = 14).	0.95
[19], England, Wales and Scotland, March to April 2008	Face to face interviews, adjusted proportions	National cancer database/general public	2872	**Age, years** 1315 (46), 16–44^ 997 (35), 45–64 564 (20), ≥ 65 **Education** 542 (19), Degree or higher 1496 (52), Below degree 837 (29), No qualifications **Ever had cancer? No** 2701 (94) **Sex, male** 1319 (46)	Confusion by medical practitioners about the need for consent to use medical data for research. The Data Protection Act (1998) allows for the use of data for medical research without consent. 82% had not heard of the registry, but 95% believed that the data it collects was important. Conflict between the current Act and organisational policies and procedures which state that assumptions cannot be made about patients being willing to have their health data shared for purposes other than for direct patient care.	0.95
[22], Belgium, February 2017	Interviews, deductive analysis using QUAGOL	Reuse of clinical trial samples and data/clinical trial participants	16	**Age, years** 35–79, mean 62, median 64 **Sex, male** 7 (43.75) **Education** 10 (62.5), higher education 6 (37.5), college or university **Ethnicity** 15 (93.5), Belgium 1 (6.25) Polish **Cancer types** 4 (25), colorectal 3 (18.75), ovarian 1 (6.25), gastric and lung 1 (6.25), colorectal and lung 2 (12.5), pancreatic 2 (12.5), gastric 1 (6.25), cholangiocarcinoma 1 (6.25), unreported	*Only results about data sharing are reported* Data was seen by participants to be a similar resource to tissue samples; however, this position is not supported legally where the samples are not considered the same. Respondent views varied on the need for re-consent prior to data access; a stratified approach may allow individual preferences to be met. • Where data is re-used by the original research team, no re-consent was needed. Respondents were supportive of an interactive consent tool where preferences could be individualised. This allows for greater control of their data. Where data is shared with an ‘unknown’ group of researchers, some wanted to be involved by re-consenting. These respondents did not object to the idea of data sharing, rather they were concerned about data security and a lack of trust.	0.9
[31], Scotland, May to June 2009	Focus groups, thematic analysis	General medicine/general public	19	**Age, years** 1 (5), < 60 15 (79), 60–74 3 (16), ≥ 75 **Numbers taken part in medical research** 6 (32) **Numbers with chronic health condition** 13 (68) **Numbers with loyalty cards** 15 (79) **Sex, male** 6 (32)	Respondents expressed a positive attitude towards medical research. • The nature of the research and who was accessing the data were important. • Respondents were surprised that anonymised data could be used in Scotland without consent. Attitudes varied on the use of anonymised health data from medical records. • A small number believed that consent was needed; this was related to the possibility that the data could never really be fully anonymised. • This is even in the setting of a one-off consent for all future use. • Most participants did not indicate the need to be informed of the data use. The process of anonymisation does not necessarily exclude the need for consent. Respondents recognised that re-consenting patients would be logistically difficult.	0.85
[33], UK, not reported	Focus group, thematic analysis	General medicine/general public	19	**Age, mean (range)** 61 (54–69) **Employment** 11 (58), employed 5 (26), retired 1 (5), unemployed seeking work 2 (11), unemployed due to illness or disability **Sex, male** 19 (100)	*Results of the qualitative focus group presented* Respondents were positive about research; a few were surprised that this research using existing data is currently undertaken. • All respondents would consent to a review of their medical records if asked. Participants were divided equally when asked about the use of medical records without prior informed consent. • Great good and public benefit versus best practice to ask. By providing information on research bias and research processes respondents became more accepting of using medical data for research without consent. • Participants were aware of difficulties of re-consent and potential low response rate which may bias findings. • Some still believed that informed consent was necessary. • Some curiosity about what research they are contributing to. Those who wished to be informed about the research, opt-out consent was acceptable. This gives the individual the right to refuse but also informs them how the data is being used. Anonymisation of data and data encryption were seen as safeguards to research with no prior consent. • A minority suggested that there are no safeguards that made them happy with a no consent model. • The role of ethics committees in patient protection was not included in respondents’ comments. The use of data and who was accessing it was important to researchers.	1
[38], USA, not reported	Focus groups, emergent content analysis	General medicine/general health	30	**Age, years** 1(3), 18–30 4 (13, 31–40 4 (13), 41–50 8 (27), 51–60 4 (13), 61–70 6 (20), 71–80 1 (3), ≥ 80 **Sex, Male** 14 (47) **Education** 11 (37), some HS 7 (23), HS 7 (23), some college 3 (10), college **Ethnicity** 4 (13), white 5 (17), black 20 (67), Latino 2 (7), other	If no consent is sought, some believed that it would be an invasion of privacy to access health data. This extended to the secondary use of this data.	1
[45], USA, 2006 and 2008	Interview (telephone and enhanced face to face), multilevel random effects logistic regression	General medicine/health and retirement study	6384	**Age, by birth cohort** 747 (11.7), < 1923 428 (6.7), 1923–1930 3543 (55.5), 1931–1941 792 (12.4), 1942–1947 875 (13.7), 1948–1953 **Sex, male** 2522 (39.5) **Ethnicity** 5235 (82.0), white 875 (13.7), black 275 (4.3), other **Education** 1481 (23.2), 0–11 years 2190 (34.3), 12 years 1334 (20.9), 13–15 years 1379 (21.6), ≥ 16 years	Overall consent rates were 67.8%. • Males (69.1%) were more likely to consent to data linkage than females (66.9%). • White respondents were more likely to consent than other racial groups (69.1% versus 65.8%). • Married respondents were more likely to consent than those separated/divorced (69.5% versus 64.9%). • No differences by age cohort or level of education. After controlling for variables only level of education was associated with consent. Respondents with a college qualification were more likely to consent compared with those with lower levels of education. Privacy and confidentiality concerns impacted the likelihood to consent. • Respondents who did not answer financial questions were less likely to provide consent. Those who are resistant to interview were also less likely to refuse consent.	1
[59], Australia, not reported	Interviews, framework approach	General medicine/general public	26	**Age, years** Between 24 and 41 **Education** 3 (12), ≤ Year 12 6 (23), TAFE 16 (62), tertiary 1 (4), post-graduate **Sex, male** 6 (23)	Respondents were supportive of data linkage for health research, particularly when it will benefit society. • Participants recognised that people have the right to refrain from participating in research using their data. Misconceptions about data linking were noted, with some believing that it is sharing of personal and health information within a healthcare system. The assumption that opt-in and specific consent was not supported. Most participants believed that data linkage research could be undertaken without consent. • Researchers should only be able to access de-identified data. • Current protections are sufficient. • Most preferred no consent over being informed of the intent to use data. The assumption that participants would prefer to provide consent for both identifiable and non-identifiable data was not supported. • Generally, most participants believed that consent is not required for data linkage. • De-identified data should not be treated the same as identifiable data. • Many believed that once identifiers were removed, the information became detached from the individual and was ‘just information’. • Not all supported this belief with some still requiring consent. • For most respondents, the focus was on data analysis, not the linking process. *Scenarios* In all scenarios presented, respondent s chose ‘no consent’ required. • The linking of de-identified health data and criminal records by experts, while acceptable without consent, while acceptable, the respondents believe that patients with mental health issues should be allowed to provide consent. • The linking of health, WorkCover and employment data linked by researchers was one scenario where consent and notification of research was required by the majority (*n* = 15). This was due to the researchers undertaking the data linkage, not an independent linking organisation. Respondents shifted their views on consent based on the scenarios presented; moving from consent required to no consent. Reasons for requiring no consent included: acceptable due to benefit, large dataset serves as protection, practical considerations, audit activities do not need consent, use of de-identified data does not breach privacy.	0.9
**Mixed methods**						
[24], England, September 2015 to December 2017	Interviews and online survey, thematic analysis	Human Fertilisation and Embryology Authority registry/fertility clinic attendees	60 (20, interview 40, online survey)	*Interview population* **Age, years** 36 median, 30–46 range **Ethnicity** 16 (80), British white **Sex, male** **5 (25)** **Occupation** 14 (70), managerial or professional 2 (10), intermediate 3 (15) routine or manual 1 (5), student	*Interview population* 14 (70) agreed to share data 2 (10) refused to share their data 3 (15) were unsure about sharing data 1 (5) agreed and disagreed with data sharing at different times *Online survey* 32 (80), agreed to share data 4 (10), refused to share their data 2 (5), were unsure about sharing data 2 (5), agreed and disagreed with data sharing at different times • Consent forms are completed while patients are under stress, and consent for use of data is seen as a low priority. • Perceived benefits and risks were associated with different levels of consent. • Increased awareness of research using routine data may increase consent rates. • Consent processes and rates vary by clinic; some patients when presented with lots of forms just tick ‘no’ to all questions. • Language was seen as a barrier to consent in patients who need interpreters. • Refusing consent if retrospective opt-out would be difficult. • Providing education and information is important to increase rates of c consent. • Information needs to clearly state the type (anonymous or identifiable) of data to be used. I*nterview* • Respondents (*n* = 20) in the higher socio-economic groups (*n* = 15) 3 refused consent, 2 were unsure about what they had agreed. • Respondents in lower socio-economic groups all consented to data use. Influences on consent included: perceived benefits and harms, trust, understanding (forms, research, anonymity), state of anxiety/being overwhelmed.	0.9
[27], USA, November 2003 to June 2004	Deliberative sessions and surveys. Quantitative-Chi squared for categorical data and ANOVA for continuous variables Qualitative- content analysis	General medicine/veterans	217	*Characteristics of deliberators provided* **Age, years—mean (SD)** 65 (12) **Education** 80 (37), BS or BA or higher **Sex, male** 206 (95)	Respondents’ willingness to share data changed based on who requested access to their data. • 34% of respondents believed that the current method of consent was OK (ethics boards decide when additional consent is required to access medical records). • 66% believed that patients should be able to tell the review board whether or not they want to share their data. • 26% would want opt-out consent. • Those who wanted opt-in consent: 35% wanted blanket authorisation; 39% wanted to be asked for consent every time.	0.9
[36], Australia, February to December 2006	Focus groups, thematic analysis Survey, chi square test of independence	General medicine/general public	723 (23, focus group 700, survey)	**Age, years (survey population)** 35 (5), 18–19 138 (19.7), 20–34 141 (20), 35–44 208 (29.7), 45–59 178 (25.4), ≥60 y **Education (survey population)** 66 (9.4), not finished HS 159 (22.7), finished HS but no HSC 131 (18.7), finished HS and HSC 17 (2.4), some technical or commercial/TAFE 24 (3.4), finished technical or commercial/TAFE 13 (1.8), some university/C.A.E. 85 (12.1), tertiary diploma 15 (2.1), now at University/C.A.E. 145 (20.7), university/C.A.E. degree 40 (5.7), post-graduate degree	Concerns relating to consent and privacy are not always connected. Opinions on obtaining consent for using health information for research varied from not important to the provision of study information. *Survey* 73% would share their health data for medical research. • 12% would not and 14% were unsure. • The majority would share sensitive data if it were not identifiable. • 92% of respondents wanted to be asked permission to use data • 83% want to know what organisation and the type of research being conducted prior to allowing access. • 5% were opposed to the idea that an individual’s permission should be sought prior to its use for a purpose other than medical treatment. • 40% of those wanting to be asked for permission were not concerned about privacy. • Therefore, 89% of those not concerned about privacy believe that permission should be sought when details used for a purpose other than medical care. The findings were similar between the focus groups and survey.	0.95
[39], England, June to July 2016	Surveys and interviews, not described	Cancer registry/Cancer patients and non-cancer patients, cancer	2033 (1033 with cancer, 1000 general public)	**Age, years, cancer group** 31 (3), 18–34 155 (15), 35–54 847 (82), ≥ 55 **Age, years, general public** 290 (29), 18–34 350 (35), 35–54 350 (35), ≥ 55 **Cancer status, cancer group only** 186 (18), localised/stable 31 (3), advanced 671 (65), remission/cancer free **Cancer type, cancer group only** 52 (5), bladder 93 (9), bowel/colorectal 258 (25), breast 134 (13), prostate 62 (6), cervical/womb 155 (15), skin 300 (29), all others **Ethnicity, white** 1002 (97), cancer group 920 (92), general public **Family or friend who has/had cancer, general public group only** 640 (64), yes **Sex, male** 475 (46), cancer group 490 (49), general public	65% of PLWC support the current data collection process compared with 52% in the general public (automatic inclusion in cancer registry at diagnosis). • 23% (PLWC) neither oppose nor support; 2% did not know. • 28% (general public) neither oppose nor support; 7% do not know. Awareness of registry changed respondents’ perceptions of the registry and data collection methods. Knowledge of opt-out option was linked with support in PLWC. 73% of respondents who think that their information can be withdrawn also supported the current method of data collection. 9% of PLWC and 12% of general public respondents oppose the current system. Of those who oppose the current data collection practices: • 50% (PLWC) and 43% (general public) of respondents believed that permission should be obtained before inclusion in the registry. • 9% (PLWC) and 6% (general public) concern about not being told about the registry. • 6% (PLWC) and 17% (general public) registry should be opt-in not opt-out. • 6% (PLWC) and 2% (general public) believed identifiable information should not be included. • 6% (PLWC) and 2% (general public) concern regarding third-party access. • 2% (PLWC) and 7% (general public) believed that the diagnosis/treatment is enough to deal with. • 10% (PLWC) and 7% (general public) did not know. Across both PLWC and general public, respondents’ concern were similar. • 50% (PLWC) and 43% (general public) were concerned about permission not being sought prior to registry inclusion. • 6% (PLWC) and 17% (general public) believed that the registry should be opt-in. PLWC who were opposed to the current data collection methods, but who supported cancer data collection more broadly, were concerned about the lack of a consent process.	0.6
[49], Australia, not reported	Focus groups and semi-structured interviews, open coding and NVivo analysis	Epidemiological research/general public and expert stakeholders	45 *(calculated based on the below)* 4 focus groups with general public (4 to 8 persons per group) 2 focus groups with Aboriginal and Torres Strait Islander peoples (4 to 8 persons per group) 5 people from diverse cultural backgrounds 20 expert stakeholders	Not reported	The process of obtaining informed consent or refusal should be regarded as the cornerstone of contemporary research ethics. Some expressed a sense of obligation about making routinely collected data available for research if government health services have been used; however, consent must be given. In contrast, this same position was used to argue that consent should not always be sought. • The individual may not recognise their obligation to society to share their data for research.	0.9
[53], Great Britain, November to December 2015	Deliberative workshops and face to face interview, not reported	Commercial access to health data/general public, doctors, individuals with chronic or rare disease	2263 (246 focus groups 2017 interviews)	Not reported	Knowing that there are safeguards in place changed the acceptability of data sharing. • 49% no safeguard • 56 to 64% where a safeguard is in place. 54% of respondents would like to be asked permission before data is shared with a commercial organisation, even if this means that the research may not proceed if permission is not granted. 53% wanted strict rules about data not being passed to third parties. Many participants changed their views on consent after discussion, moving from consent being needed every time to seeing that this may be impractical. • By increasing education around research and data use, respondents may not wish to opt-out. Participants wanted more information on aggregation and anonymisation. They noted a lack of consistency more generally about opting-in and opting-out of things generally in society. If consent cannot be sought for all access to data, there should be clear communication why this is. Consent can help reassure the participant that they have control on their data.	0.95
[56], Canada, not reported	Public dialogues and survey, ANOVA and MANOVA	General medicine/general public	98	**Age, years** 37 (38), 20–39 35 (36), 40–59 26 (27), ≥ 60 **Education** 26 (26), HS or less 18 (18.7), some post-secondary 42 (42.7), completed post-secondary 12 (12.5), post graduate or professional degree **Sex, male** 40 (40.8)	Broad opt-in consent for the use of personal information was the favoured option in the presented scenarios. • Consent for each use and assumed consent were equally represented across the spectrum. Where profit was part of the scenario, respondents moved to consent processes with greater control. • Personal control was the commonly cited approach for improving the individual’s confidence in the use of their information for research. No one method of consent was acceptable to all respondents and different types should be developed for different types of research. • Individuals should be able to see who has accessed their record for non-clinical care. Aspects for each consent method were liked by respondents. • Consent for each use was an opportunity for education, increased communication, and a better understanding of the individual’s role in research. • This research was seen to respect the individual’s privacy. • Respondents acknowledge this process is burdensome for the research team and may lead to sampling biases. • Assumed consent was seen as the most efficient approach and the least subject to bias. Reduced burden on the individual needing to give consent. • Places onus on the individual to opt-out if they do not want to participate. • Lack of individual education and control with this approach. • Broad consent was seen as a compromise between consent for each use and assumed consent. • Less burdensome than consent for each use, but as an opt-in approach, offered individual control. Ability to control access was a key theme; respondents wanted the opportunity to opt-out. Concern about control over data once released to researchers was expressed. *Scenarios* 1. *Using prescribing information for quality improvement*: 21% happy for data to be used without notification; 27% notification with opt-out option. One-fifth opted for broad consent and 25% wanted to be asked for permission for each use. 2. *Using prescribing information for market research*: ~60% believed information should not be used for this purpose. 14% believed permission should be sought each time; only 5% opted for passive use without notification; 13% notification with opt-out. 3. *Linking work, education, or income with health information*: 10% believed this information should not be linked; ~ 25% preferred that permission be sought each use; 17% use of data with data; 17% use of data with notification and opt-out process. 4. *Linking of health information with leftover tissue*: non-commercial access—similar response to scenario 3. 5. *Linking of health information with leftover tissue*: commercial access (for profit): 45% permission required for each use; 18% no linkage should occur at all. The use of data safeguards made respondents more receptive to research using data.	0.5
[57], Canada, November 2006 to July 2007 and September 2007	Survey and focus groups, regression analysis using generalised estimating equations	General medicine/DM, HT, chronic depression, alcoholism, HIV, BC, LC and general public.	1780	*(n* = *1137)* **Age, mean** 54 years **Sex, Male** (43) **Education** 33), HS or less **Self-described health** **3**3), poor or very poor 40), fair 27), good or excellent	*Completed survey n* = *403* Consent preferences were similar between health conditions. Research involving profit or linking of health information to education, income or occupation records required more restrictive consent processes. • Respondents were more willing to link biological samples with health information that with education, income, or occupation records. Consent preferences ranged from ‘Just use it’ to ‘information should not be shared’. The majority believed the following, rates were similar between the general public and target health condition populations. • Just use it was seen as appropriate for quality improvement linking of data and biological sample where no profit is made. • Information used for marketing and for profit were not widely supported. • Permission to use data before each use was required when information was linked to biological samples for profit or when it was liked with information on education, income, or occupation. Those more concerned with privacy required more restrictive consent processes. Use of data for profit was conditional on more restrictive consent processes. No consent option was supported by a majority of respondents making it difficult to propose a single method for the secondary use of health data.	0.9
**Other**						
[35], USA, 1997	Letter requesting consent, percentage of respondents, logistic regression	General medicine/attendees to medical centre	2463	**Age, years** 618 (25.1), 20–39 624 (25.3), 40–59 612 (24.8), 61–79 609 (24.7), ≥ 80 **Sex, male** 1233 (50.1)	3.2% declined authorisation (95% CI, 2.4% to 4.0%) access to medical records for research. • If those who did not respond to request are also considered to have refused, 20.7% (95% CI, 18.5% to 22.9%). Women were more likely to refuse authorisation compared with males (4% and 2.4% respectively, *p=*0.067). Patients under the age of 60 years were also more likely to refuse compared with older patients (5.4% compared with 1.2% respectively, *p* < 0.001)*.* Respondents over 120 miles (193 kilometres) from the study centre were more likely to authorise access compared with local respondents (2.1% versus 5.8% respectively, *p=*0.001). Respondents with a *more sensitive* diagnoses such as mental health conditions, infectious diseases or parasitic disease, and reproductive issues) were less likely authorise access.	0.9
[43], New Zealand, not reported	Citizens jury, not applicable	Pharmacoepidemiology research/general public	13	**Age, years** 18–65 (7 (54) were 45) **Sex, male** 6 (46)	The jury agreed that researchers contracted by a public body should be permitted to access medical records with identifiable information only in some circumstances. • Routine analysis to identify potential adverse effects from newly introduced medicines. • Investigating emergency concerns about the adverse effects of medicines currently being used. Several safeguards were established to protect the use of the data. Most jury members were happy for their medical information (identified solely by their medical record number) to be used for research and to improve the health of New Zealanders.	1
[51], UK, not reported	Citizens jury, not applicable	General medicine/general public	34	**Age, years** 8 (23.5), 18–29 10 (29.4), 30–44 10 (29.4), 45–59 6 (17.6), ≥ 60 **Sex, male** 17 (50) **Education** 13 (38.2), no qualification 11 (32.4), apprenticeship or other qualification 10 (29.4), degree level or above	33 (97.1) were in favour of secondary use of data for research. *Methods of consent* • 24 (70.6), opt-out. • 6 (17.6), opt-in. • 3 (8.8), all records should be available • 1 (2.9), no change. Public benefit was a justification for access. Views on data sharing changed over the jury process. 17(50), became more willing to share for public benefit. 2 (5.9), opted for more control.	0.8
[52], Ireland, 2007	Letter requesting consent, not described	General medicine/general public	1178	**Age, years** 50.9 (SD 20.8) (mean), 47 (median) **Sex, male** 37 (20.1%)	14.5% (*n* = 171) of respondents explicitly opted out of the research. • 142 by letter. • 15 by website. • 2 by phone. • 12 returned letters without indication. Those who opted out via the website were younger compared with those who responded via letter (nonparametric, 53.5 years compared with 38.7 years, *p* < 0.05). Patients who opted out were slightly older (52.8 years versus 50.4 years; not statistically significant) and were more likely to be female (83% versus 79.5%; not statistically significant). 1.9% (*n* = 22) opted into the research. 2 patients expressed concern regarding the opt-out methodology and how their contact details were obtained. 83.4% participated indicating that opt-out consent was and acceptable method of recruitment.	0.9
[60], USA, January 1997 or February 1997	Verbal request to sign consent form, descriptive statistics, Mantel-Haenszel test, and multivariate logistic regression	General medicine/general public	15,997	**Age, years** 4384 (27), ≤ 16^ 5892 (37), 17 to 40^ 4059 (26), 41 to 64 1662 (10), ≥ 65 **Sex, male** 6490 (41)	90.6% of participants granted authorisation to use data from their medical records for research. • 3.6% refused authorisation. • 4.5% were undecided. • 1.3% were not asked (for example those receiving emergency care). Refusal rates were higher in respondents who received care for mental health concerns, trauma, or eye care. Those aged 39 years and older were also more likely to refuse. The highest rates of undecided were in women presenting for pregnancy care.	0.8
[61], Australia, March to December 1997	Letter requesting consent, not reported	General medicine/participants in the ALSWH	39,883	**Age, years** 14,228 (36), 18–23 13,338 (33.4), 45–50 12,317 (31), 70–75 **Sex, male** 0 (0)	49.4% provided consent for record linkage: 37% of the young women, 59% (*n* = 7898), of the mid-age women and 53% of the older women. Consenters in all age groups had higher levels of education and were more likely to have private health insurance. Similar proportions of consenters and non-consenters in the young and mid-age groups experienced major personal illness and hospitalisation in the previous year.	0.8

*ADL* activities of daily living, *AIDS* acquired immune deficiency syndrome, *ALSPAC* Avon Longitudinal Study of Parents and Children, *ALSWH* Australian Longitudinal Study on Women’s Health, *BC* breast cancer, *BHPS* British Household Panel Survey, *CAE* Centre for Adult Education, *CATI* computer assisted telephone interviewing, *CC* colon cancer, *CF* cystic fibrosis, *CI* confidence interval, *DM* diabetes mellitus, *ED* emergency department, *GCSES* General Certificate of Secondary Education, *GED* general educational development, *HCP* healthcare professionals, *HIV* human immunodeficiency virus, *HKID* Hong Kong Identity Card number, *HS* high school, *HSC* high School certificate, *IAB* Institute for Employment Research, *IRB* institutional review board, *IQR* interquartile range, *LidA* Leben in der Arbeit, *MS* multiple sclerosis, *NHI* National Health Insurance, *NHS* National Health Service, *NILT* Northern Ireland Life and Times, *NZ* New Zealand, *OR* odds ratio, *SCD* sickle cell disease, *SHI* statutory health insurance fund, *PLWC* people living with cancer, *PS* primary school, *SD* standard deviation, *SF-36* 36-item health survey, *SS* secondary school, *TAFE* Technical and Further Education, *VA* Veterans Affairs, *UK* United Kingdom, *USA* United States of America

Adults or parents of children with CF, or adults or parents of children with SCD, or adults or parents of children with DM, or adults with HIV, or adults with BC, or adults with CC

#Only participants born in 1959 and 1965 were recruited to this study

^Only participants over 18 were included in the systematic literature review protocol; data presented in the study included respondents aged between under 18 years of age. For completeness, the data has been included in the demographics section of this table

### Study design, location, clinical focus and study populations

Qualitative research methodologies included interviews (face-to-face or via telephone) [16, 19, 22, 45] and focus groups [31, 33, 36, 38], a combination of both [49] or surveys and focus groups [57]. Other methodologies included questionnaire-based interviews [50], surveys [15, 17, 18, 20, 21, 25, 26, 28–30, 32, 34, 37, 39, 40, 42, 44, 46, 48, 53–55, 58, 59] and combinations of deliberative sessions/interviews and surveys [23–25, 27, 56, 57]. Two studies used a citizens’ jury model [43, 51], one study was a randomised controlled trial [47], one was a nested cohort within a randomised controlled study [41], three studies requested consent to access data by letter [35, 52, 61] and one requested consent directly from the participant [60]. Studies were conducted in several countries; a breakdown by country is presented in Table 3.

**Table 3:** Studies by country

Country study undertaken (in alphabetical order)	Number of studies	Reference
Australia	6	[21, 36, 48, 49, 59, 61]
Belgium	1	[22]
Canada	6	[15, 32, 42, 55–57]
England	2	[24, 39]
Europe	2	[25, 28]
Finland	1	[30]
Hong Kong	1	[41]
Ireland	1	[52]
Germany	1	[50]
Great Britain	2	[23, 53]
New Zealand	2	[43, 54]
Northern Ireland	1	[44]
Scotland	1	[31]
Taiwan	1	[34]
UK	9	[16, 19, 26, 29, 33, 37, 46, 47, 51]
USA	10	[17, 18, 20, 27, 35, 38, 40, 45, 58, 60]

Most articles focused on the attitudes of the general public towards the access to and secondary use of digital health data in different settings, particularly general medicine [16, 18, 20, 21, 26, 30, 31, 34–38, 40, 41, 44–48, 50–56, 58–61], but also National Cancer databases [19, 39], and pharmacoepidemiological [43] and epidemiological [29, 33, 49] research. Other studies focused on the attitudes of health consumers to secondary data analysis and sharing of health data in individuals: attending US Veterans Affairs (VA) facilities [27], retirees [45], recently discharged from tertiary care [23], patients attending a hospital with a cancer diagnosis [32], cardiology patients [15] and patients with asthma and stable angina [17]. Other articles focused on responses from those with acquired immune deficiency syndrome (AIDS) or multiple sclerosis (MS) or mental health concerns [42], rare diseases [25, 28], clinical trial data [22], fertility [24] and respondents with potentially stigmatising conditions (DM, hypertension, chronic depression, human immunodeficiency virus [HIV], breast cancer or lung cancer) [57].

Articles considered general attitudes towards health data use [15–18, 20, 21, 25, 26, 37, 38, 41, 45–48, 50, 57–59, 61], linking health administrative data to clinical trial data [32] and reuse of clinical trial data [22], access to medical records [23, 27, 29, 31, 33, 35, 40, 42–44, 48, 51, 52, 54–58, 60], statistical databases [36], registries [19, 24, 28, 30, 39], health data for epidemiological research [49] and the linking of health insurance data with survey data [34]. Commercial access to health data [53] was considered in one article. Five studies included data from respondents both over and under the age of 18 years of age [16, 19, 26, 37, 60]; where this has happened, only data from respondents over 18 years of age have been included in this analysis.

### Mechanisms of consent

Several mechanisms of consent were discussed in the articles; a description of each of the consent mechanisms, by study, is included in Table 4.

**Table 4:** Types of consent and brief description

Type of consent	Description
Assumed	Consent to use data is not sought, as it is believed that the individual would agree to its use if consent were sought.
Broad (or blanket)	The individual provides consent for a broad range of research activities. This consent may be for certain activities, such as research within a particular disease, or for all research purposes.
Dynamic consent	The consent process is iterative and can change with the individuals changing preferences about how they want their data to be used. This process also provides information to the individual about research more generally and the results of previous research using the data.
No consent required	Data which can be used without specific consent from an individual.
No consent with notification	Where explicit consent is not sought, but the individual is advised that their data is being used for a specific research purpose.
Opt-in	The consent process requires the study participant to actively agree to participate in a study.
Opt-out	The consent process requires the study participant to actively exclude themselves from study participation.
Passive consent	Consent is assumed where an individual does not decline to participate.
Retrospective consent	Consent to use data is obtained after the data has been collected.

### Study quality

Results of the quality assessment are provided in Table 2. QualSyst [12] scores ranged from 0.35 to 1.0 (possible range 0.0 to 1.0). While no studies were blinded, most provided clear information on respondent selection and data analysis methods and used justifiable study designs and methodologies. No key themes stood out for studies which were deemed to be of lower quality. No data were from randomised studies; the highest level of evidence was from a nested cohort, with other data obtained from studies which used surveys and interviews.

## Consent preferences

The included studies described several consent models including opt-in, opt-out, passive, broad (blanket), none, assumed and dynamic models (see Table 4). Key observations from the included studies are presented below and in Table 2.

### Studies from Continental Europe

Five studies described the issues of data usage and consent preferences of Continental European respondents [22, 25, 28, 30, 50] representing a total of 6790 respondents. Consent preferences for accessing data held in registries were not universal, and a willingness to share health data was not precluded by a wish to control access to it [25]. In the setting of registry-based research, respondents to a quantitative study were divided between believing that a single informed consent was acceptable, that no consent was necessary, that consent should be sought in some cases or sought every time [30]. Some respondents believed that their data should not be used at all for research, while others believed that everyone’s medical records should be available for research [30]. Where there is a potential for the data to be used for research, respondents wanted to be informed at the time of hospital admission, and over half of respondents wanted specific details about the type of research for which it could be used [30]. In the setting of a leukodystrophy registry, respondents in a qualitative study believed that a combination of initial broad consent, supported by ongoing information about the use of data, research results and new partnerships (academic and/or pharmaceutical companies) was acceptable [28]. The use of an interactive consent tool, where individuals could specify their consent preferences and exercise more control over their data, was supported [22].

### Studies from the UK and Ireland

Sixteen studies [16, 19, 23, 24, 26, 29, 31, 33, 37, 39, 44, 46, 47, 51–53] from the UK and Ireland discussed health data usage and consent preferences representing a total of 108,824 respondents. The proportion of respondents who would give permission to access information in their medical notes was high, but varied based on the type of information to be used [23]. Most respondents wanted to be asked when data relating to side effects and medical history were taken from medical records, while few wanted to be informed when data on sex and age were sought (5% and 7% respectively) [23]. Signing a consent form to allow future data use during the patient’s hospitalisation was a preferred method but respondents were also happy to be informed, but not required to provide consent, each time the data was to be used [23]. Variation in views on the need for consent was seen in other studies; while some respondents acknowledged that re-consent is difficult and has the potential for low response rates, they still saw it as best practice to ask [33]. For some, an opt-out consent process was seen as acceptable as it allows the individual the right to refuse participation while informing them of the types of research being undertaken [33, 51, 52]. Higher rates of support were shown for the current data collection methods used for the UK cancer registry in people living with cancer (PLWC) (65%) compared with general public respondents (52%); however, 9% (PLWC) and 12% (general public) were opposed [39]. Of those who were opposed, 6% (PLWC) and 17% (general public) believed that it should be an ‘opt-in’ process rather than ‘opt-out’ [39]. While there are opt-out and data withdrawal options for the UK cancer registry, few were aware of these options [39]. Two studies noted the complexity of retrospectively opting out of data collection processes [24, 31]. A citizens’ jury process found that respondents preferred opt-out consent over opt-in (70.6% vs 17.6%); a small percentage (8.8%) believed that all records should be available for use for the public benefit [51]. In one study, younger respondents equated an ‘opt-in’ process with being asked if their data could be used [16].

### Studies from North America

Ten studies from the USA [17, 18, 20, 27, 35, 38, 40, 45, 58, 60] and six from Canada [15, 32, 42, 55–57] discussed secondary data use and consent preferences representing a total of 42,620 respondents. Studies from Canada showed variability in consent preferences and included the use of consent each time, broad consent, and notification with opt-out consent [55]. Many respondents believed that no consent or notification was required in some circumstances [42, 55]. Where general consent was provided, 80% wanted to periodically review this decision, while others wanted either no or minimal involvement (36%) or notification with an opt-out option (24%) [55]. Various consent processes were proposed across the studies and included opt-out [27], opt-in [56], opt-in/one-off consent with blanket authorisation [27, 42], consent to be sought each time [27, 42, 56], assumed consent [56] or no requirement for consent but with individuals being informed about data usage [42]. Respondents liked different aspects of each consent process [56]. For some, where consent was not sought, respondents considered it to be a violation of privacy [38]. In the setting of potentially stigmatising health conditions, respondents to a mixed-methods study had a range of views about consent ranging from ‘just use it’ to ‘the information should not be shared at all’ [57]. Respondents felt that where data were to be used for quality improvement, no consent was needed; however, where data were to be linked or used in profit-based activities, consent was required before each use [57].

### Studies from Australia and New Zealand

Six studies from Australia [21, 36, 48, 49, 59, 61] and two from New Zealand [43, 54] discussed consent preferences and represented a total of 42,104 respondents. While seeking consent to use data should be the ‘cornerstone’ of contemporary research practice [49], views on consent about the use of health data for research ranged from not important to the need to provide detailed information [36]. Respondents to a qualitative study believed that while people had the right to refuse access to health data for research, most believed that data linkage research could be undertaken without consent [59]. Reasons for not requiring consent included a perceived benefit from research, large datasets serving as protection, practical considerations, that the use of de-identified data does not breach privacy, and that audit activities do not require consent [59]. Where data was to be used for epidemiological research, respondents in a mixed-methods study were divided about the requirement for consent [49]. Opt-out consent was considered acceptable to 54% of the respondents in one study, but nearly one-third (28%) preferred an opt-in method and a fifth had no preference (19%) [21]. The use of a hybrid consent model was proposed in a quantitative study from New Zealand [54]. This method proposed a mix of general consent processes including specific criteria for use in the context of accessing data in the clinical setting and the requirement for specific consent when data is accessed for other purposes [54].

Several other themes were addressed in the included papers and are presented below by theme.

## Support for medical research and permission to access data

Several studies noted broad support for medical research [15, 19, 28, 30–33, 36, 42–44, 49, 51, 55, 58, 59] using both identifiable [42] and de-identified or anonymous [31, 36, 43] data. Reasons for support included the potential to develop/improve medical care and reduce medical errors [42], develop new treatments [25], and an increased understanding of disease and diagnosis [25]. Other reasons included a sense of obligation to make data collected when using government health services available [49], and to benefit society more generally [44, 59]. A small percentage (approximately 4%) of respondents were not supportive of using medical data for research [28, 30, 55]. In an Australian study, 46% of respondents assumed that their health data was already used for medical research, without explicit consent [21], and in a second study, respondents believed that health data was a similar resource to tissue samples [22]. Several articles discussed preferences in relation to the levels of permission required to access data and varied by the type of data to be accessed. Support for secondary data usage was high, in one study, 97.4% of respondents (*N* = 590) would allow researchers to access their data for the purposes of conducting a clinical trial [15] or for health services research (67%; *N* = 1,106) [58]. However, a significant number of participants did not provide consent, between 10% (*N* = 3,429) [17] and 25% [58]. Respondents wanted to be asked permission when their data was to be used for reasons other than treatment [36], but permission was least required when age or sex was extracted [23]. Even in the setting of using anonymous data, respondents wanted to or preferred to be asked permission [23]. Some respondents believed no consent was required prior to use when their health data were anonymous [36, 42]. Where consent was not feasible, but required to conduct the research, over 50% of respondents believed that it was essential, even if it meant not conducting the research [42]. Nearly half of the respondents (50% PLWC, 43% general public) were concerned about a lack of permission being sought prior to their data being included in the UK cancer registry [39].

## Record linkage

Agreement with record linkage was generally high [18, 20, 26, 34, 37, 50] ranging between 96.3% (*N* = 2,271) [18] and 86.2% (*N* = 1,574) [20]; however, agreement for data linkage was significantly lower in other studies [61], with rates between 41% (*N* = 6,433 and *N* = 13,454) [37, 46] and 67.8% (*N* = 6384) [45]. There was some misunderstanding about what data linking is, with some believing that it relates to information sharing within the health system [59]. A lack of consensus on the requirement for consent for data linkage is evident. Some respondents wanted to provide consent [16, 44], broad consent [56], consent in specific circumstances [30], or notification with opt-out options [56]. Some respondents believed that there was no requirement for consent [16, 44, 56, 59], while others believed that everyone’s data should be available for research [30]. In one study, if consent was not obtained, 31% of respondents (*N* = 1,202) believed that the research should be abandoned; however, an equal number believed that while consent should be sought, the research should not be abandoned if it was not possible (34%) [44].

Consent for record linkage was influenced by health status [20], socio-economic variables [26], demographics [20, 26], and what datasets were to be linked [56]. Where data was linked to education or economic data [46, 55] or a government identification number (ID) [41] agreement for record linkage decreased. Respondents who provided no information on income were less likely to give consent to data linkage, if they did consent it was more likely for administrative data [50]. Linking health data to education, income or occupation records, resulted in respondents wanting more control over consent mechanisms. Interestingly, respondents were more willing to link health data to biological samples, compared with this data, although consent was required [57].

## Organisations and individuals conducting the research and proposed use of data

Consent to share data was influenced by the organisations or individuals conducting the research and the nature of the data to be shared [23, 36, 54]. In the setting of health registries, there was support for the information to be used for aetiological studies, disease monitoring, and assessing the effectiveness of healthcare; many believed that the information should be used for any research purpose [30]. In one quantitative study, there was strong support for using data when used for rare disease research (90%); however, when the data was used for research into other diseases, the rate of consent was lower [28]. The intended use of data made little impact on support for data reuse, with many having no consent preferences or preferring not to be asked [23]. Respondents preferred to have their data extracted by either a doctor’s nurse [55] or by the NHS [44], although in both studies, some respondents believed both options to be unacceptable. Respondents were less willing to share data with government agencies [42, 54], employers [42], health insurers [54], pharmaceutical companies [42], or with commercial organisations [53]. In these settings, respondents wanted to provide consent [42, 53, 56], even if this means that a lack of consent would stop the research proceeding [53]. Where profit was involved, respondents moved to consent processes with greater control, and where data was to be linked for market research or with leftover tissue specimens, high rates of either ‘should not be used’ or ‘permission required each use’ were seen [56]. For others, the use of data for marketing and/or profit was not seen as appropriate [57]. Where data were to be shared with an outside organisation, the need for re-consent was higher, however this was due to issues of trust and data security more than an objection to data sharing [22]. In the setting of a rare disease, 60% of respondents (*n* = 149 relatives, *n* = 46 patients) were willing to have registry data used for research undertaken in partnership with pharmaceutical organisations; about 10% were opposed to this [28].

## Identifiable versus non-identifiable/de-identifiable data

Differences in acceptability of the use of health data varied by its identifiability and individual preference. Respondents believed that where data is to be used in an identifiable manner, this needs to be clearly stated at the consent stage, and where data were non-identifiable, respondents were more willing to allow access without consent [24, 42]. Studies from Australia [59] and New Zealand [43, 54] found that respondents were more willing to share de-identified data compared with identifiable data. Respondents believed that identifiable data should only be accessible to researchers in specific circumstances such as to ensure medicine safety [43]. Some respondents believed that once patient identifiers had been removed, it becomes detached from the individual and is just information which should be available to researchers [59]. Even when anonymous data are to be used, some voice concern about sharing this data with non-healthcare professionals [54]. Some respondents believed that the process of anonymisation did not remove the need for consent; this was due to concerns about the effectiveness of data anonymisation processes [16] and the belief that data could not ever be fully anonymised [31]. Some respondents believed that the process of anonymisation and data encryption were safeguards, and therefore, no consent was required to use the data [33].

## Influences on consent

### Age

Several studies found differences in attitudes towards consent by respondent age [17, 30, 52]. Across the studies, older respondents were more willing to share or link data than younger participants [20, 32, 46] and those with younger respondents were more willing to consent to health data use [25, 35, 37, 58, 61] and/or record linkage [26, 34]. One study found that while patterns of consent were similar across responders, consent decreased with age (over 50 years of age) in both males and females [29].

### Sex

Differences were seen in support for data reuse and methods of appropriate consent processes by sex; however, these were not always significant [26, 30, 46, 52]. Where it was a factor, males were more likely to consent to medical record review [29] and believed that informed consent was not required for registry-based research more than women [30]. Women believed that consent should be required in some cases more than males (44% compared with 31%) [30], were less likely to give authorisation [35], and consented less [58] suggesting a greater desire for control over their data [25] compared with males.

### Location

By location, respondents from Northern Ireland (adjusted OR 50.56; 95% CI, 0.50–0.63) had lower levels of support for data access compared with respondents from other parts of the UK (adjusted OR 51.17; 95% CI, 1.06–1.29) (*N* = 50,994) [26], and respondents from the former West Germany were less likely to consent to data linkage compared with those from Berlin or East Germany [50]. Respondents living closer to a study centre (5.8% and 2.1%) had higher rates of refusal compared with those living further away [35].

### Education and socio-economics

Levels of education influenced attitudes towards consent. Respondents with a secondary education were more likely to provide consent compared with those with lower levels of education [25, 26, 37, 45, 46, 50, 58, 61]. Education level also impacted a respondent’s willingness to share health data without consent. Those with no academic qualifications were less likely to agree to research on health data being conducted without consent compared with those with higher levels of education (38%, no qualification; 26%, graduate level), with this decreasing with as the respondents’ level of education increased [44]. Those with lower levels of education and who had lower incomes were less likely to be non-responders to requests for consent compared with respondents who actively declined consent [58]. In one study, respondents who were married, illiterate, who lived in a suburban area (compared with urban/rural) or those with a lower monthly income were less likely to consent to data linkage [34]. Three studies noted the impact of socio-economic variables on consent; however, all reported different outcomes. While one study found that socio-economics did not influence consent [37], other studies found that low income did influence consent patterns [34] and that respondents in the higher socio-economic group were less likely to provide consent when compared with those in lower socio-economic groups [24]. Respondents who declined to answer questions about income from investments had lower levels of consent for data linkage, however other socio-economic variables did not appear to influence consent [46]. Those who provide consent were more likely to have health insurance [61]. Other influences on providing consent for data use included undertaking voluntary work or previous data record linkage by a family member [46].

### Ethnicity

Differences in consent preferences were noted by respondent ethnicity. Research from a study in the UK found that consent for data linkage was significantly lower (58.9%) in respondents (*N* = 50,994) who identified as non-white compared with those who identified as white (72%) [26]. This was similar to two other studies from the UK and the USA where those who reported their ethnicity to be British/Irish white had higher consent compared with other groups [37], and those who identified as being of African-American heritage were less likely to consent to data usage [58]. Language and the need for an interpreter were also seen to influence consent. In an Australian study, opt-out consent was preferred by respondents who were illiterate in their primary language or who were refugees [21] while an English study found language to be a barrier to discussing/seeking consent [24].

### Health status

Consent preferences were found to be influenced by the respondent’s health status. Respondents with lower physical and mental functioning scores, measured using the 36 Item short form (SF-36) questionnaire, were shown to have lower levels of consent for data usage [34]; however, in a second study, respondents with lower SF-36 physical and mental health scores were more likely to consent [58]. In one quantitative study, respondents with lower Charlson Comorbidity Index (CCI) scores were less likely to allow survey and health data linkage compared with respondents with higher scores [20]. Experience of a major personal illness and hospitalisation in the last year [61] and severity of illness [58] did not impact the frequency of consent; however, respondents with more severe rare diseases were more likely to support data sharing compared with those with less severe diseases [25]. Consent was higher in respondents with life-limiting and lifelong illness [26], those with health conditions under investigation [29], those with two or more medical diseases [50], those in good health (compared with those in poorer health) [30], and those with diabetes or who were obese [37]. In contrast, in one quantitative study respondents with long-term health conditions were more likely to be against health data sharing compared with those with no long-term health conditions (~ 25% vs 20%) [44]. Respondents (*N* = 15,997) who presented for mental health concerns, trauma, and eye care had higher rates of refusal for providing access to medical records for research [60].

### Sensitive topics

Several health topics were considered more sensitive than others and influenced consent preferences [48]; the need for ethical oversight when research is undertaken on a sensitive topic [30] was noted. Respondents were less likely to provide consent to access medical records for research which included sensitive topics [35]. Requesting data on sexual orientation [23], sexual or sexually transmissible diseases [42], infectious diseases [35], reproductive issues [35], contraception or female genital disorders [58], urinary disorders [58], mental health [25, 35, 42], disability [25] and genetics [25] were all reasons for increased need to ask permission or provide consent to access data or were associated with lower levels of consent. Interestingly, in one study non-consent rates were higher in respondents seeking care for uncomplicated diabetes and or headache [58], conditions which may not be considered sensitive by healthcare consumers. Women receiving pregnancy care had the highest response of ‘undecided’ about providing consent [60]. In the setting of potentially stigmatising conditions (HIV, chronic depression, HT, alcoholism, DM, breast or lung cancer), provision of consent was similar between diseases [57]. The diagnosis of stable angina or asthma was not considered particularly sensitive [17]; however, for others, information about symptoms and the name of their disease [25] was. Consent was lower in respondents who did not answer questions on smoking status, income, or functional status [58], however, questions relating to income did not always decrease consent [47]. Respondents to a study from New Zealand were less willing to share data of a sensitive nature with those not involved in their care [54]. In one study, respondents with HIV, MS or mental health presentations believed that only physicians involved in their care should be able to access medical records without consent [42]. Where data was to be linked, respondents in one study were unable to reach a consensus on consent when medical data were to be linked with data on teenage pregnancy and state benefits, birthweight and future health outcomes, mental health and criminal records, and asthma diagnosis and postcodes [16]. In other studies, some respondents identified the need for consent where health, employment and WorkCover (workers compensation insurance) data [59] were linked. Consent was not required when health data were linked to cancer registry and death data, and if de-identified health data and criminal records [59].

### Other

Several articles discuss the relationship between trust, transparency, privacy and consent, and quality, and these issues are explored at length in a subsequent publication [9]. Other influences on consent included health literacy and government insurance status [18], or requesting a national identity number [41]. Other negative influences on consent included a fear of discrimination, fraud, threat to personal safety and use of data without their knowledge or in a context they do not approve of [25]. Further, perceived benefits and harms, trust, anxiety or feelings of being overwhelmed, and understanding of processes and research more generally influenced consent [24]. Where data were to be used in a for-profit setting or where individuals were more concerned about privacy [57] and where information was to be shared with a third party [25] respondents required more conditions and more restrictive consent processes. Interestingly, in a Canadian study, there was a difference in consent rates for the use of paper-based versus electronic records, with 27% percent of respondents (*N* = 1,235) believing that electronic data can be used without permission compared with 12% for paper records [55]. Respondents who responded to the request for consent via a website were younger compared with those who responded by letter (38.7 years compared with 53.5 years) [52].

### Ethics committees and institutional review boards

The role of human research ethics committees (HREC) and institutional review boards (IRBs) in influencing the need for consent was noted in one study [40]. In one article, the requirements of the IRB were shown to have significantly impacted the overall rates of study consent and completion [40]. Three studies [25, 27, 42] described respondents’ views about the role of HRECs in deciding if researchers should be allowed to access existing health data for research (secondary data analysis). Even in the setting of HREC approval and where the research was deemed low harm, 67.7% of respondents (*N* = 235) wanted to be asked to consent to the use of their data for medical research [42]. This was similar to a European study, where near equal numbers of respondents (*N* = 2,013) were for (49%) and against (43%) allowing an HREC to decide about access to data [25]. Finally, one study found that a third of respondents believed that the decision of the HREC regarding consent was sufficient, although two-thirds of respondents wanted to be able to tell the HREC how they wanted their data shared [27].

## Activities to improve acceptance of data use

Several studies noted inconsistencies in respondent expectations about secondary data use and local requirements (laws, policies, procedures), with some not requiring individual patient consent to use data [32]. In the UK, the legal framework allows for the use of data for medical research without consent [19]; however, there is also a requirement for individual consent when individual-level administrative data is to be linked to research such as survey data [46]. Inconsistencies between UK legislation and institutional policies and procedures can cause confusion [19]. In one Finnish study, a similar proportion of respondents argued for both a tightening of legislation and an increased liberalisation of laws relating to health data (35% versus 25%) [30]. The inconsistent application of consent generally in society, with both opt-in and opt-out being used, can be confusing to participants [53].

Education and the provision of more information about data use and research practices were cited in several articles. Researchers need to clearly communicate how an individual’s data is to be used and at the conclusion of the study provide feedback and the study results to the participant [25]. Other activities, such as providing increased information about their disease, the capacity to withdraw consent, and being informed of any data breaches if they occur, also promote acceptance of secondary data use in health [25]. Where respondents were provided with information on research processes, they became more accepting of the use of medical data without consent [33, 53]. The requirement for consent was connected to an individual’s desire to control access to data [56], and where obtaining consent is not practical, for transparency researchers should clearly communicate to participants why this is so [53]. Further, individuals should be clearly informed of current data sharing practices [54, 59] when receiving health services; one study noted that implied consent is not always well-informed consent [54]. Increasing the awareness of health research using routine data will increase the acceptability of this type of research and may increase rates of consent [24]. Requesting consent when individuals are under stress or anxious can result in fewer individuals who consent [24]; an Australian study found a delay in seeking approval to link health and survey data did not compromise authorisation rates [48]. In a citizens’ jury process, 50% of respondents (*N* = 34) became more willing to share their health data for public benefit, a small percentage (5.9%) opted for more control over their data after receiving education and information on research using health data [51]. Clear rules about data not being accessible to third parties and knowledge of these safeguards can increase the acceptability of data sharing [53, 56].

## Discussion

This systematic literature review highlights the ongoing complexity associated with secondary data analysis and linking of health data. While respondents believed that the principles of data sharing were sound, agreement on the type and need for consent was not universal. In many circumstances, individuals may not be aware of the current national laws and regulations which govern health data sharing and linking in their countries, and these may be inconsistent with their personal beliefs on the need for consent. The variation in responses about the type of consent that individuals prefer suggests two things. First, that the use of a single type of consent process for all research projects may not be appropriate and that mechanisms such as dynamic or hybrid models should be considered. Second, that even in the setting of seeking consent to use data, not all individuals will agree with consent processes used, or potentially with the use of secondary use of health data more generally.

Differences in the need for consent are complicated by the identifiability and subject of health data to be used. While respondents were generally happy to consider sharing their health data for research, de-identified or anonymous data appeared to be more acceptable to many respondents. Differences in consent preferences require researchers to be conscious of variations by age, sex, ethnicity, location, and education levels of their potential research participants, as this may affect the level of consent preferred. Further, the type and subject matter of the data used may also impact attitudes towards consent, and researchers should be conscious that topics of a sensitive nature may attract less consent. The role of ethics committees in determining the need for consent in studies using secondary data analysis should consistently reflect the requirements of national and local laws and regulations. Researchers need to be better informed about the requirements for consent when undertaking research using health data. Finally, education and information should be provided to the health consumer about data protection mechanisms, how an individual’s health data can and will be used and by whom, and the laws and policies governing secondary use of health information.

## Limitations

The papers included in this study were limited to those indexed on major databases, some literature on this topic may have been excluded if it was not identified during the grey literature and hand searching phases. As the search was restricted to English language publications, some relevant literature may have been excluded from the search. We note that health systems and health funding models may be different in non-English speaking countries and that Western ideals of autonomy may not be universally held.

## Implications

Results of this systematic literature review indicate that respondents recognised the advantages of health research using existing health data and are generally supportive of these initiatives. The provision of increased information to individuals about data protection and data usage is central to the use of health data for research. Therefore, health organisations and those who act as data custodians should work towards increasing the awareness of current data sharing practices and data protections in their patient populations.

## Conclusion

The literature confirms that individuals are generally supportive of using health data for research, particularly in a de-identified or anonymous format. By increasing the awareness of current data sharing practices and data protections in individuals who interact with the health system, the use of medical records for health research may become more widely accepted as a regular function of medical care.

## Data Availability

All data generated or analysed during this study are included in this published article.
